# Cinaciguat Prevents Postnatal Closure of Ductus Arteriosus by Vasodilation and Anti-remodeling in Neonatal Rats

**DOI:** 10.3389/fphys.2021.661171

**Published:** 2021-07-29

**Authors:** Yu-Chi Hung, Yi-Ching Liu, Bin-Nan Wu, Jwu-Lai Yeh, Jong-Hau Hsu

**Affiliations:** ^1^Graduate Institute of Medicine, College of Medicine, Kaohsiung Medical University, Kaohsiung, Taiwan; ^2^Department of Pediatrics, St. Joseph Hospital, Kaohsiung, Taiwan; ^3^Department of Pediatrics, Kaohsiung Medical University Hospital, Kaohsiung Medical University, Kaohsiung, Taiwan; ^4^Department of Pharmacology, College of Medicine, Kaohsiung Medical University, Kaohsiung, Taiwan; ^5^Department of Medical Research, Kaohsiung Medical University, Kaohsiung, Taiwan; ^6^Department of Marine Biotechnology and Resources, National Sun Yat-sen University, Kaohsiung, Taiwan; ^7^Department of Pediatrics, School of Medicine, College of Medicine, Kaohsiung Medical University, Kaohsiung, Taiwan

**Keywords:** ductus arteriosus, soluble guanylyl cyclase, remodeling, vasoconstriction, vascular smooth muscle

## Abstract

Closure of the ductus arteriosus (DA) involves vasoconstriction and vascular remodeling. Cinaciguat, a soluble guanylyl cyclase (sGC) activator, was reported with vasodilatory and anti-remodeling effects on pulmonary hypertensive vessels. However, its effects on DA are not understood. Therefore, we investigated whether cinaciguat regulated DA patency and examined its underlying mechanisms. *In vivo*, we found that cinaciguat (10 mg/kg, i.p. at birth) prevented DA closure at 2 h after birth with luminal patency and attenuated intimal thickening. These anti-remodeling effects were associated with enhanced expression of cyclic guanosine monophosphate (cGMP) in DA. *Ex vivo*, cinaciguat dilated oxygen-induced DA constriction dose-dependently. Such vasodilatory effect was blunted by KT-5823, a PKG inhibitor. In DA smooth muscle cells (DASMCs), we further showed that cinaciguat inhibited angiotensin II (Ang II)-induced proliferation and migration of DASMCs. In addition, cinaciguat inhibited Ang II-induced mitochondrial reactive oxygen species (ROS) production. Finally, Ang II-activated MAPKs and Akt were also inhibited by cinaciguat. In conclusion, cinaciguat prevents postnatal DA closure by vasodilation and anti-remodeling through the cGMP/PKG pathway. The mechanisms underlying anti-remodeling effects include anti-proliferation and anti-migration, with attenuation of mitochondrial ROS production, MAPKs, and Akt signaling. Thus, this study implicates that sGC activation may be a promising novel strategy to regulate DA patency.

## Introduction

Ductus arteriosus (DA) is a vital vessel connecting the pulmonary arteries and aorta during fetal period and mostly will be closed soon after birth. Patent DA is the major cause of co-morbidities in preterm newborn and, on the other hand, is life-saving in newborns with ductus-dependent congenital heart disease (Hamrick and Hansmann, 2010). Thus, the mechanisms of regulating DA patency are important issues in the field of vascular research.

Ductus arteriosus closure involves two phases: functional and anatomic closure (Bokenkamp et al., 2010). DA constriction causes functional closure within hours after birth, and subsequent vascular remodeling results in permanent anatomic closure. After birth, increased oxygen tension and declined prostaglandin E_2_ (PGE_2_) are two major factors for DA constriction. Subsequent DA remodeling is composed of complex histological changes, such as extracellular matrix deposition, and intimal thickening caused by proliferation and migration of smooth muscle cells (SMCs).

Current medications for regulating DA patency is mainly mediated by the PG pathway, with cyclic adenosine monophosphate (cAMP) as the second messenger (Yokoyama et al., 2013). For medical closure of the PDA in preterm newborns with heart failure, PG synthetase inhibitors such as indomethacin or ibuprofen are widely used. For treating newborns with ductus-dependent congenital heart defects (CHDs), PGE_1_ is the only choice to keep the DA patent for maintaining pulmonary circulation. However, both PG synthetase inhibitors and PGE_1_ have several significant adverse events (Lewis et al., 1981; Oncel and Erdeve, 2015). Therefore, developing a novel pharmacological target is essential for newborns with PDAs and ductus-dependent CHDs.

Cinaciguat, a soluble guanylyl cyclase (sGC) activator, could activate heme-deficient sGC to increase intracellular cyclic guanosine monophosphate (cGMP) for further biological effects and is currently used in patients with pulmonary hypertension (Evgenov et al., 2006). Previous studies have shown that cinaciguat declined pulmonary pressure and resistance because of vasodilation (Evgenov et al., 2007; Chester et al., 2011) and inhibited vascular remodeling through anti-proliferation and anti-migration of vascular smooth muscle cells (VSMCs) (Dumitrascu et al., 2006; Hirschberg et al., 2013). However, there is no study investigating whether cinaciguat could prevent DA closure.

In this study, we hypothesize that cinaciguat could prevent postnatal DA closure through vasodilatory and anti-remodeling effects on neonatal rats. We also explored the molecular mechanisms underlying these effects on DA smooth muscle cells (DASMCs) treated with angiotensin II (Ang II), a mediator implicated in DA closure (Crockett et al., 2019; Hamrick et al., 2020).

## Materials and Methods

### Animals

Timed pregnant Wistar rats were purchased from BioLASCO Taiwan Co., Ltd. (Taipei, Taiwan). The DA was obtained from a newborn rat on the 21st gestational day (full term). This study was approved by the Animal Care and Use Committee of the Kaohsiung Medical University. The Institutional Animal Care and Use Committee (IACUC) number is 102094. The animals were cared for in compliance with the guiding principles of the National Institutes of Health of the United States.

### Reagents

Cinaciguat was purchased from MedChemExpress (Princeton, NJ, United States). Ang II, β-actin and α-smooth muscle actin antibodies, and MTT were obtained from Sigma-Aldrich Inc. (St. Louis, MO, United States). MitoSOX was purchased from Molecular Probes (Eugene, OR, United States). Antibodies against ERK1/2, Akt, and antibodies against phosphorylated ERK, JNK, Akt were obtained from Cell Signaling Technology (Beverly, MA, United States). Antibodies against JNK was obtained from R&D Systems (Minneapolis, MN, United States). Antibodies against p38 were purchased from Santa Cruz Biotechnology, Inc. (Dallas, TX, United States). Antibodies against phosphorylated p38 were obtained from Abcam (Cambridge, United Kingdom). Dulbecco’s Modified Eagle Medium (DMEM), FBS, penicillin, streptomycin, and all other tissue culture reagents were purchased from Gibco BRL Life Technologies (Grand Island, NY, United States).

### Morphological Analysis of DA Closure

To determine whether cinaciguat could prevent DA closure *in vivo*, newborn rats were injected intraperitoneally with cinaciguat (10 mg/kg) in the cinaciguat group or saline (10 ml/kg) in the control group immediately following spontaneous delivery by the pregnant rat on the 21st gestational day (full term). After injection, the neonates were incubated in room air at 33°C. Half of the rats in each group were sacrificed at two time points, 0 or 2 h after injection, by decapitation followed by opening the chest wall. The morphology of the DA and main pulmonary artery (PA) was observed under a dissecting microscope to measure the external diameter of DA and PA. For determining the degree of DA closure, we calculated the ratio of DA/PA.

### Histological Analysis of Luminal Patency and Intimal Thickening

Following morphological analysis, the DA was taken down, and the sectioned segments from the middle portion of the DA were analyzed histochemically. Paraffin-embedded blocks containing these tissues were cut into 3-μm-thick sections and placed on glass slides. We used Orcein stain to reveal the internal elastic lamina, the boundary line between the intima and media layers of the DA, for further determining intimal thickening and medial area of the DA. The intimal thickening and luminal area were estimated by (intimal area)/(medial area) and (luminal area)/(total vessel area), respectively, as previously described (Yeh et al., 2018). The average of at least three sections was used as the value for each tissue.

### Immunofluorescence of DA

Frozen sections of DA were cut into 10-μm-thick sections and placed on glass slides and mounted on appropriately labeled slides. Immunofluorescence staining for the target proteins were performed as described (Liou et al., 2020). The sections were incubated with 10% paraformaldehyde solution. After washout in PBS plus 0.05% Triton X-100, the sections were blocked for 30 min with 5% BSA and then incubated with rabbit antibodies against cGMP (1:100 dilution; Bioss Inc., ACD, Newark, CA, United States) overnight at 4°C. Then, the sections were incubated with a secondary Goat Anti-Rabbit IgG Antibody (H + L) FITC conjugate (dilution 1:100; Sigma-Aldrich, St. Louis, MO, United States) at room temperature. The sections were mounted and viewed with a confocal laser-scanning microscope. The average of at least three sections was used as the value for each tissue.

### Isometric Tension of the DA Vascular Rings

To determine whether cinaciguat could inhibit oxygen-induced constriction *ex vivo*, newborn rats were killed by decapitation to harvest the DA ring immediately after spontaneous delivery by pregnant rats on the 21st gestational day (full term). The DA was placed in a tissue bath and kept at 37°C. Two stainless steel wires (40-μm internal diameter) were threaded into the lumen, and the preparation was mounted in a four-channel myograph (Multi-Wire Myograph System 620M, Aarhus, Denmark). One stainless steel wire was connected to a micromanipulator, and the other was connected to a force transducer. All the DA rings were initially stabilized for at least 60 min with a modified Krebs–Henseleit solution, which was equilibrated under hypoxia (0% O_2_, 5% CO_2_, and 95% N_2_) and maintained at 37°C by a heated water jacket. The isometric tension was continuously monitored using a PowerLab 4/26 system (AD Instruments, Inc., Colorado Springs, CO, United States), as previously described (Yeh et al., 2018). All the vascular rings were stabilized and relaxed, with the resting tension adjusted to 5 mN. DA preconstriction was attained by hyperoxia exposure (95% O_2_ and 5% CO_2_). The vasodilatory effect of cinaciguat (1–100 nM) was assessed at peak O_2_ constriction by the application of incremental doses at 5-min intervals. To determine the role of the PKG pathway, we examined the effects of cinaciguat on O_2_-induced DA contraction by pretreatment with KT5823 (10 μM), a PKG inhibitor, 10 min before hyperoxia exposure. At the end of the experiments, vasoconstriction of the DA was induced by KCl (50 mM)-containing Krebs–Henseleit solution to assure vessel reactivity.

### Primary Culture of Rat DASMCs

DA smooth muscle cells in primary culture were all obtained from the DA of newborn Wistar rats as previous described (Yeh et al., 2018). In brief, immediately after spontaneous delivery by the pregnant rat on the 21st gestational day, the newborn rats were killed by decapitation followed by taking down the DA. The outer sphere of the DA was peeled, and the intimal layer of DA was shaved lightly two to three times to remove endothelial cells. DAs were then plated onto Petri dishes (Corning Inc., Corning, NY, United States) and cultured in DMEM supplemented with 10% FBS, 100 U/ml of penicillin, and 100 μg/ml streptomycin, maintained in a humidified incubator (37°C, 5% CO_2_, and 95% air). When the cultures reached confluence, cells were subcultured using 0.5% trypsin. The purity of the DASMCs was confirmed under a confocal microscope by immunofluorescence staining for α-smooth muscle actin (>95% of cells stained positive), with a typical “hill and valley” appearance and lack of staining for vimentin, as previously described (Wu et al., 2016). Serum starvation before the experiments was achieved by 1% FBS for 48 h to synchronize cells into quiescence.

### Cell Proliferation Assay

To assess DASMC proliferation, an MTT assay was first performed as previously described (Hsu et al., 2011). In brief, after serum starvation for 48 h, proliferation of DASMCs was induced by Ang II (10-nM) for 48 h in DMEM supplemented with 1% FBS in 96-well plates at a density of 4 × 10^4^ cells per well, with or without pretreatment (1 h) and co-treatment (48 h) with cinaciguat (0.1–10 μM). After induction for 48 h, MTT (0.5 mg/ml) was added to the medium for 4 h. The culture medium was then removed, and the cells were dissolved in isopropanol and shaken for 10 min. The amount of MTT formazan was quantified at absorbances of 540 and 630 nm using an enzyme-linked immunosorbent assay (ELISA) reader (Dynex Technologies, Denkendorf, Germany). In addition, the DNA synthesis of DASMC was also assessed by the bromodeoxyuridine (BrdU) incorporation assay (Roche Applied Science, Mannheim, Germany). The DASMCs were seeded into 96-well plates at a density of 4 × 10^4^ cells per well, and the confluent DASMCs were induced by Ang II (10 nM) with or without cinaciguat (0–10 μM) for 24 h in the same medium containing BrdU (10 μM). Cells were harvested for the detection of DNA synthesis by incorporation of BrdU using a cell proliferation ELISA. The DASMCs were measured colorimetrically using a Bio-Rad model 680 μL plate reader (Bio-Rad, Hercules, CA, United States) at a wavelength of 450 nm.

### Cell Migration Assay

Migration of DASMCs was assessed on Transwell polyethylene terephthalate cell culture inserts with 8-μm pores as previously described (Hsu et al., 2011). After serum starvation for 48 h, DASMCs (2 × 10^4^ cells) were loaded into the upper compartment and incubated for 48 h at 37°C (5% CO_2_ and 95% air). Meanwhile, in the lower compartment, Ang II (10 nM) was dissolved in DMEM, with or without pretreatment (1 h) and co-treatment (48 h) with cinaciguat (0.1–10 μM). After 48 h, the non-migrated cells on the upper membrane surface were removed, and those on the lower surface were fixed in methanol and stained with Giemsa. The number of cells per six high-power fields (200 × HPF) was counted, and the mean number of cells was used to express migration activity.

### Measurement of Mitochondrial ROS Production

Mitochondrial reactive oxygen species (ROS) production in DASMCs was measured using MitoSOX, a fluorescent probe specific for mitochondrial superoxide. After serum starvation for 48 h, DASMCs (1 × 10^5^ cells) were stimulated by Ang II (10 nM) for 48 h with or without pretreatment with cinaciguat (0.1–10 μM). MitoSOX (5 μM, molecular probes) was added for a 10-min incubation period at room temperature and was fixed by 1% formaldehyde after washing with PBS. Cellular fluorescence was examined under a fluorescence microscope at an excitation/emission of 510/580 nm following the instructions of the manufacturer. Results were expressed as fluorescence intensities in arbitrary units (AU).

### Western Blot Analysis

The protein samples were extracted from the whole cells following methods previously described (Liou et al., 2004). The extracted protein solutions were stored at -80°C until analysis. Equivalent amounts of protein were resolved by SDS-polyacrylamide gel electrophoresis (PAGE) (10–14%) and transferred to polyvinylidene difluoride membranes. After blocking for 1 h in 5% non-fat dry milk and Tris-buffered saline, the membrane was incubated with the desired primary antibody for 2 h. The membrane was then treated with the appropriate horseradish peroxidase-conjugated secondary antibody (Chemicon Inc., Temecula, CA, United States), and the immunoreactive bands were detected by enhanced chemiluminescence (ECL) reagents (PerkinElmer Life Sciences Inc., Waltham, MA, United States). Proteins labeled with specific primary and secondary antibodies were visualized with enhanced chemiluminescence. β-Actin was probed as a control to ensure equal protein loading.

### Statistical Analysis

Results are expressed as the mean ± the standard error (SEM). In the *in vivo* study, the statistical interactions between DA diameter, luminal patency, intimal thickening, and cinaciguat treatment were obtained by two-way ANOVA or unpaired *t*-test. In the DA tension study, repeated two-way ANOVA was performed to compare the group and time effect. In the *in vitro* experiments, statistical differences were determined by the one-way ANOVA analysis with Bonferroni’s *post hoc* test. The Kolmogorov–Smirnov test was performed to examine the normality. A *P* < 0.05 was considered statistically significant.

## Results

### Cinaciguat Prevented DA Closure at 2 h After Birth

**FIGURE 1:**
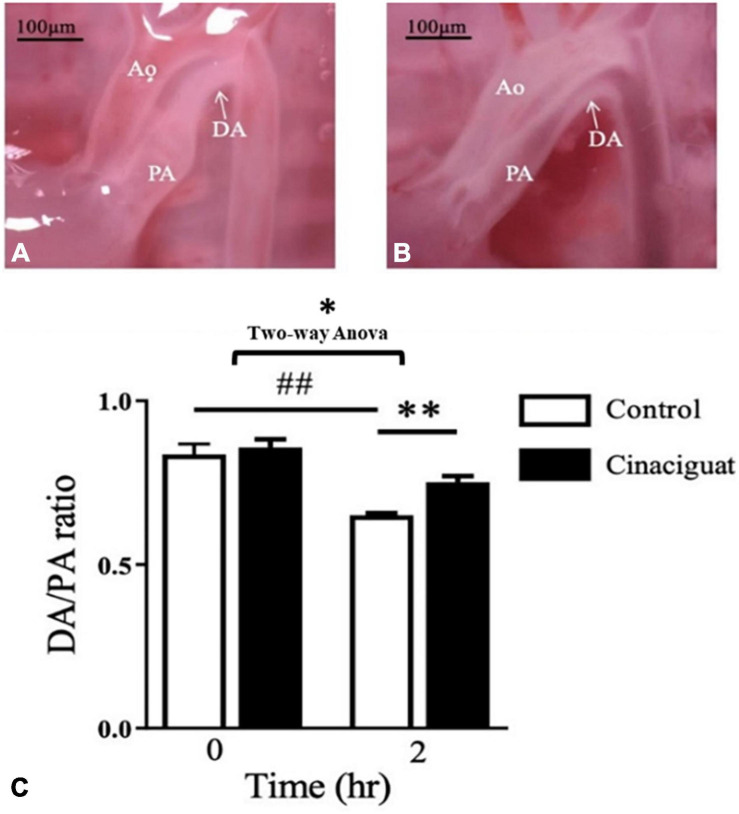
Cinaciguat prevented postnatal ductus arteriosus (DA) closure in neonatal rats. **(A)** Under dissecting microscope, we found that the DA of control rats closed at 2 h after birth with luminal obliteration. **(B)** In cinaciguat-injected rats, cinaciguat (10 mg/kg, i.p. at 0 h after birth) prevented DA closure with luminal patency at 2 h after birth. **(C)** The bar graph showed serial changes in the DA/pulmonary artery (PA) ratio in both groups. In the control group, the ratio declined significantly within 2 h after birth. Comparing control at 2 h after birth, cinaciguat group had higher DA/PA ratio. Values represent the mean ± SEM; *N* = 6. Ao, aorta; PA, main pulmonary artery; DA, ductus arteriosus. *P < 0.05 by the two-way ANOVA analysis; ^##^ and ** *P* < 0.01 by the *post hoc* test; magnification 10×.

To examine the effect of cinaciguat on neonatal rat, we injected cinaciguat into the newborn rats intraperitoneally at 0 h after birth and then examined the DA condition at 2 h after birth for comparison with control rats. We used the external diameter ratio of DA/PA to represent the degree of DA closure. In the control rats, the rats at 2 h after birth had luminal obliteration (Figure 1A) and lower DA/PA ratio compared with those at 0 h after birth (65.1 ± 0.6 vs. 83.7 ± 3.1%, ***P*** < 0.01) (Figure 1C). At 2 h after birth, the cinaciguat-injected rats had more patent DA (Figure 1B) and higher DA/PA ratio compared with control rats at 2 h after birth (75.2 ± 1.9 vs. 65.1 ± 0.6***%***, ***P*** < 0.01) (Figure 1C).

### Cinaciguat Attenuated Intimal Thickening and Maintained Luminal Patency of the DA at 2 h After Birth

**FIGURE 2:**
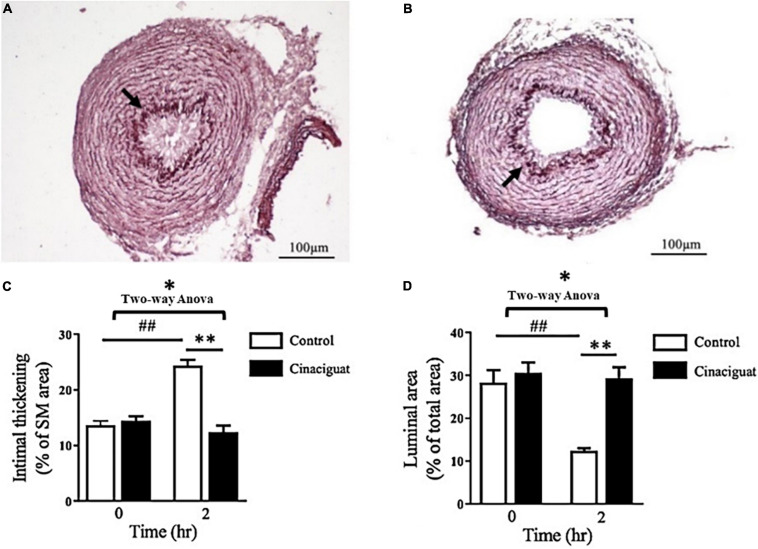
Cinaciguat attenuated intimal thickening and maintained luminal patency of the ductus arteriosus (DA) in neonatal rats. **(A,B)** Histochemical analysis of DA section showed less intimal thickening and more luminal area in the **(B)** cinaciguat group compared with the **(A)** control group at 2 h after birth. Arrows indicate the internal elastic lamina separating the intimal layer from the medial layer. **(C,D)** The bar graphs show serial changes in the **(C)** intimal thickening and **(D)** luminal area in both groups. **(C)** In the control group, the intimal thickening (ratio of intima area/media area) increased significantly at 2 h compared with 0 h, while cinaciguat attenuated such increment at 2 h. **(D)** In the control group, the luminal patency (ratio of luminal area/total vessel area) was attenuated significantly at 2 h compared with 0 h, while cinaciguat preserved luminal patency at 2 h. SM, smooth muscle. Values represent the mean ± SEM; *N* = 6. *P < 0.05 by the two-way ANOVA analysis; ^##^ and ** *P* < 0.01 by the *post hoc* test; magnification 200×.

We investigated the histological change in the DA and analyzed the effect of cinaciguat on intimal thickening and luminal area. In the control rats at 2 h after birth, we found that the lumen of the DA was obstructed with intimal thickening (Figure 2A). In the cinaciguated-injected rats at 2 h after birth, the lumen of the DA remained patent with minimal intimal thickening (Figure 2B). Quantitatively, we found that cinaciguat attenuated intimal formation as evidenced by ratio of intimal area/medial area (12.4 ± 1.2 vs. 24.4 ± 1%, *P* < 0.01) (Figure 2C) and maintained luminal patency (ratio of luminal area/total vessel area) (29.3 ± 2.6 vs. 12.4 ± 0.6%, *P* < 0.01) (Figure 2D).

### Cinaciguat Preserved Expression of cGMP While Maintaining DA Patency

**FIGURE 3:**
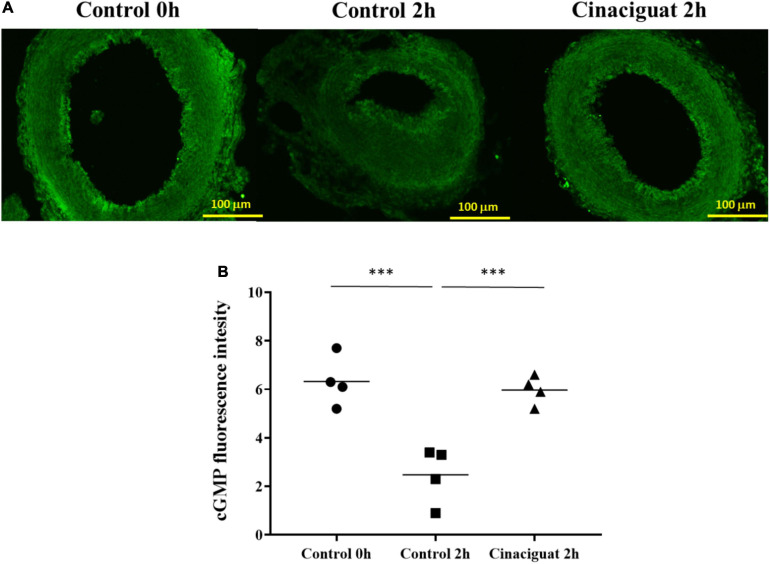
Cinaciguat prevented ductus arteriosus (DA) closure and intimal thickening with associated preservation of cGMP in neonatal rats. **(A)** Representative immunofluorescence images of cGMP. **(B)** cGMP expression levels in DA in all groups. Values are presented by the dot plot with the mean. *N* = 4. ANOVA *P* < 0.05; ***P < 0.001; magnification 200×.

The cGMP (cyclic guanosine 3′,5′-monophosphate) is an important second messenger in the NO-sGC pathway. It has been previously found that, after birth, there is an oxygen-induced decrease in intracellular cGMP levels in DA (Crockett et al., 2019). To evaluate the cGMP expression in rat DA tissue, we performed immunofluorescence analysis and image quantitation of cGMP in DA sections. At 2 h after birth, we found that there was a significant decrease in cGMP expression in the control group, but not in the cinaciguat-treated neonatal rats (Figure 3), indicating that cinaciguat prevented DA closure with associated up-expression of cGMP.

### Cinaciguat Dilated Oxygen-Induced DA Constriction

**FIGURE 4:**
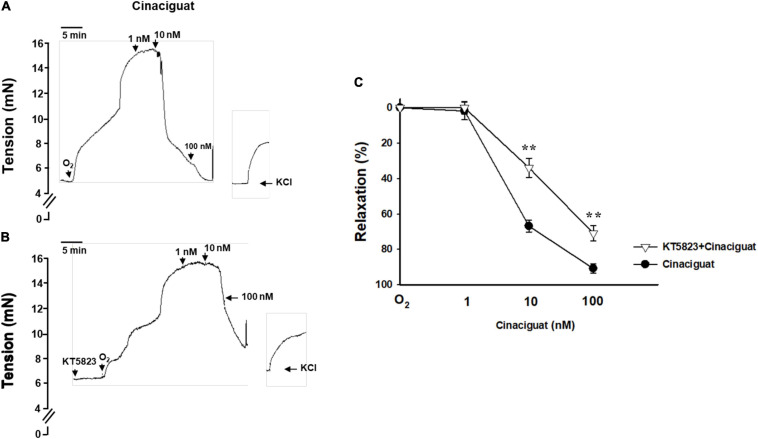
Cinaciguat attenuated oxygen-induced ductus arteriosus (DA) constriction in the myograph study. **(A)** Representative force myograph traces showing isometric tension (mN) plotted against time. After DA was pre-constricted by oxygen, addition of cinaciguat caused decrement in the DA tension dose-dependently. At the end of the experiments, vasoconstriction of the DA was induced by KCl (50 mM)-containing Krebs–Henseleit solution to ascertain vessel reactivity. **(B)** Cinaciguat blunted oxygen-induced DA constriction dose-dependently. When pretreated with KT5823 (10 μM), a PKG inhibitor, the vasodilatory effects of cinaciguat were attenuated at both 10 and 100 nM. **(C)** A comparative graph of vasodilatory effects between the two groups. Values represent the mean ± SEM; *N* = 4. *P* < 0.05 for both dose and drug effects by the repeated two-way ANOVA. **P < 0.01 compared with the cinaciguat group by the *post hoc* test.

During DA closure, vasoconstriction is an important mechanism besides intimal thickening. Given that oxygen is the main factor for DA constriction, we explored the effect of cinaciguat on the DA ring with an oxygen-rich solution. Cinaciguat declined the vascular tone of oxygen-constricted DA in a dose-dependent manner (Figure 4A). The vascular tone enhanced by oxygen was significantly attenuated by cinaciguat at concentrations of 10 and 100 nM (66.2 ± 3.3 and 90.9 ± 2.6%, respectively, ANOVA *P* < 0.01). In order to identify the underlying vasodilatory mechanism of cinaciguat, we further pretreated the DA ring with a PKG inhibitor (KT-5823, 10 μM) (Figure 4B) and found that PKG inhibition attenuated the vasodilatory effects of cinaciguat by 32 and 20% at concentrations of 10 and 100 nM, respectively (both *P* < 0.01) (Figure 4C).

### Cinaciguat Inhibited Ang II-Induced DASMC Proliferation and Migration

**FIGURE 5:**
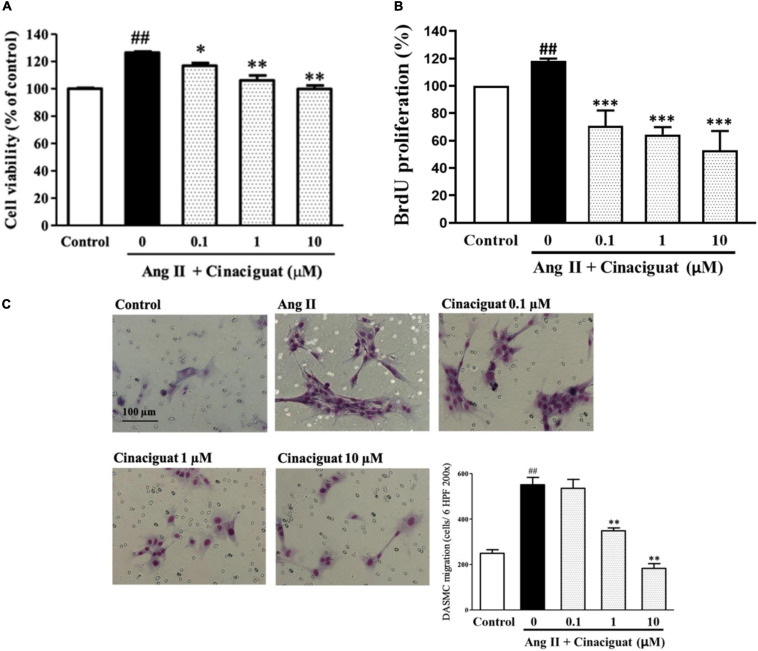
Cinaciguat inhibited Ang II-induced ductus arteriosus smooth muscle cell (DASMC) proliferation and migration. **(A)** The cell viability was augmented by Ang II after 48 h of incubation. Cinaciguat significantly inhibited Ang II-induced proliferation in a dose dependent manner. **(B)** BrdU incorporation of DASMCs was increased after incubation with Ang II for 48 h. However, cinaciguat attenuated Ang II-induced BrdU incorporation in a concentration-dependent manner. **(C)** Cinaciguat significantly inhibited Ang II-induced migration in a dose dependent manner. The photomicrograph showed the Ang II-induced migration of DASMCs from upper to lower chamber. The bar graph shows migration activity assessed by the number of cells observed in six high-power fields. Values represent the mean ± SEM; *N* = 6. All one-way ANOVA *P* < 0.0001. ^##^*P* < 0.01 vs. control; *P < 0.05, **P < 0.01, and ***P < 0.001 vs. Ang II alone (magnification 200×).

DA smooth muscle cell proliferation and migration constitute intimal thickening for DA closure. Histologically, we found that cinaciguat attenuated intimal thickening with anti-remodeling effect, then we investigated underlying cellular and molecular mechanisms via *in vitro* studies using DASMCs. To determine whether cinaciguat attenuates Ang II-induced DASMC proliferation, we first examined the viability of DASMCs by the MTT assay. The results showed that cinaciguat attenuated Ang II-induced proliferation of DASMCs in a concentration-dependent manner (Figure 5A). In addition to the MTT assay, we performed quantitative colorimetric assay for DNA synthesis based on BrdU incorporation, to examine the antiproliferative effects of cinaciguat. In parallel, we found that cinaciguat attenuated Ang II-induced DNA synthesis of DASMCs dose-dependently (Figure 5B). Furthermore, we determined the effects of cinaciguat on DASMC migration induced by Ang II (10 nM) with the Boyden chamber assay. We found that Ang II potently stimulated DASMCs but co-treatment of cinaciguat inhibited Ang II-stimulated migration in a concentration-dependent manner (Figure 5C).

### Cinaciguat Inhibited Ang II-Induced Mitochondrial ROS Production

**FIGURE 6:**
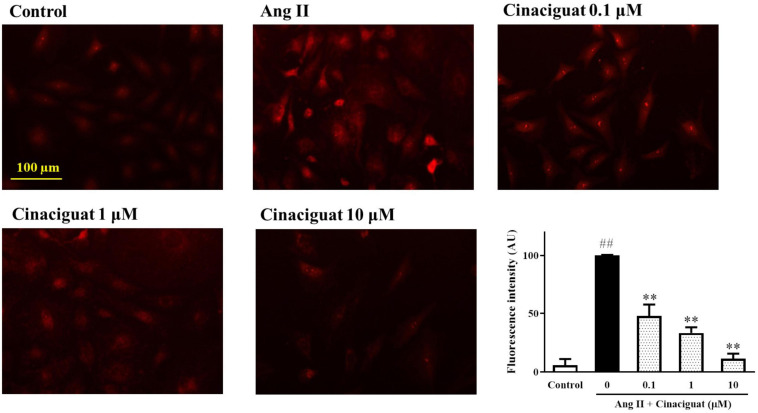
Cinaciguat-inhibited Ang II-induced mitochondrial reactive oxygen species (ROS) production. The fluorescent photomicrograph showed the mitochondrial ROS (red color) in ductus arteriosus smooth muscle cells (DASMCs). Ang II induced ROS production, but when co-treated with cinaciguat, the ROS production declined. The bar graph showed the fluorescence intensity of ROS in different doses. Values represent the mean ± SEM; *N* = 6. One-way ANOVA *P* < 0.0001. ^##^*P* < 0.01 vs. control; **P < 0.01 vs. Ang II alone (magnification 200×).

We used DASMCs to study the mitochondrial ROS production, which may have roles in mediating SMC proliferation and migration. We found that cinaciguat inhibited Ang II-induced mitochondrial ROS production dose-dependently (Figure 6).

### Cinaciguat Inhibited Ang II-Induced Phosphorylation of ERK 1/2, JNK, and p38

**FIGURE 7:**
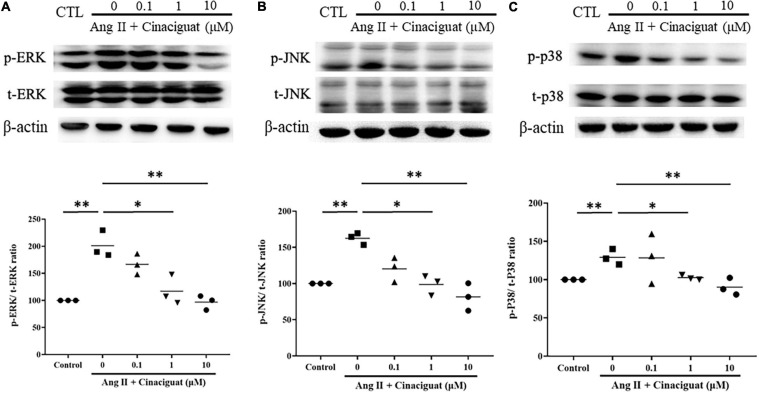
Effects of cinaciguat on Ang II-induced activations of MAPKs pathways presented by Western blotting. Western blotting shows that cinaciguat-attenuated Ang II-induced phosphorylation of **(A)** ERK 1/2, **(B)** JNK, and **(C)** p38. Values are presented by the dot plot with the mean. *N* = 3. All one-way ANOVA *P* < 0.0001. *P < 0.05 and **P < 0.01 vs. Ang II alone.

Next, we examined the effects of cinaciguat on important signaling in cell proliferation and migration, MAPK signaling including ERK 1/2, JNK, and p38. Cinaciguat attenuated Ang II-induced phosphorylation of ERK 1/2, JNK, and p38 in a dose-dependent manner (Figure 7).

### Cinaciguat Inhibited Ang II-Induced Phosphorylation of Akt

**FIGURE 8:**
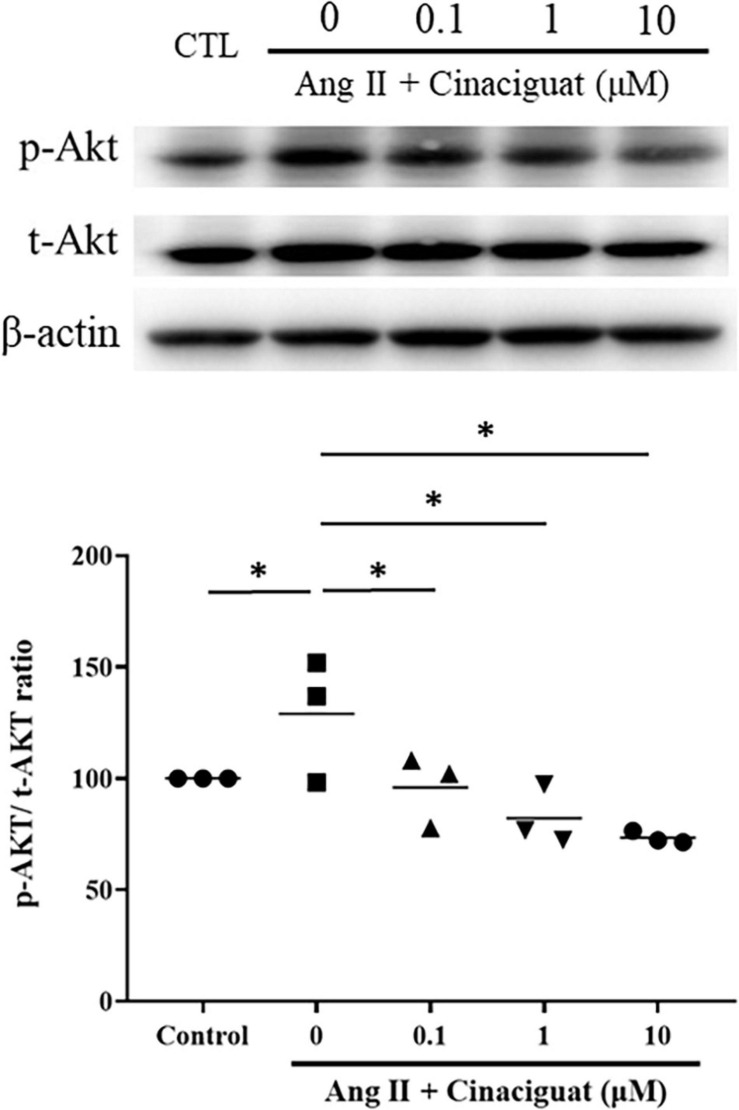
Cinaciguat-inhibited activation of Akt signaling as shown by Western blotting. Ang II activated Akt signaling significantly. Cinaciguat down-regulated Ang II-induced Akt activation dose-dependently. Values are presented by the dot plot with the mean. *N* = 3. One-way ANOVA *P* < 0.0001; *P < 0.05 vs. Ang II alone.

We also examined the effect of cinaciguat on Akt signaling, which is important for cell proliferation and migration. Cinaciguat attenuated Ang II-induced phosphorylation of Akt in a dose-dependent manner (Figure 8).

## Discussion

In this study, we found for the first time that cinaciguat, a sGC activator, could prevent DA closure in neonatal rats through vasodilatory and anti-remodeling effects. These effects were mediated, at least partly, by the cGMP/PKG pathway. To elucidate the underlying molecular mechanisms, we further demonstrated that in DASMCs cinaciguat conveyed anti-proliferative and anti-migratory effects, with attenuation of mitochondrial ROS and down-regulations of MAPK and Akt signaling. The findings indicate that sGC activation may be a novel pathway to be explored and shed some light in expanding the current pharmacological management of DA patency or DA-dependent heart diseases.

Mechanisms underlying the regulation of DA patency are complex and not fully understood (Hung et al., 2018; Lin et al., 2020). Even though the current clinical management mainly targets the prostaglandin pathway, issues such as adverse effects and incomplete responses have been unsolved (Hsu et al., 2010). Therefore, research regarding pathways beyond that of prostaglandin may have clinical implications in DA regulation. Indeed, it has been recently suggested that the NO pathway may have a role in both vasodilatation and anti-remodeling in DA (Hsu et al., 2021). For example, during DA functional closure, the genetic expression of NO synthase (NOS), a key enzyme-producing NO, was found to be significantly altered in the DA (Goyal et al., 2016). In addition, endothelial NOS, the most abundant type of NOS detected in the ductal wall, exerted vasodilatory effect on the DA (Clyman et al., 1998; Ovali, 2020). Beyond the role of NO in functional closure, it has been demonstrated in premature baboons that NO can also attenuate anatomical remodeling of the DA (Seidner et al., 2001). In vascular SMCs, it is known that sGC is the target enzyme of NO. However, tolerance or resistance to NO may limit sGC activity and cGMP production in SMCs, thus hindering vasodilation (Lakshminrusimha et al., 2016). In addition, it is known that there is a postnatal physiologic decrease in intracellular cGMP levels of the DA (Crockett et al., 2019). Therefore, a strategy of sGC/cGMP activation might be a rational approach in the regulation of DA patency after birth.

Emerging evidence shows that sGC activator cinaciguat can decrease pulmonary pressure through vasodilatory effect and has also inhibited vascular remodeling in studies on pulmonary hypertension. For example, cinaciguat caused pulmonary vasodilation in fetal lambs with chronic intrauterine pulmonary hypertension due to DA ligation (Chester et al., 2011). Similarly, in animals with pulmonary hypertension, cinaciguat attenuated muscularization of pulmonary arteries, suggesting its anti-remodeling effect (Dumitrascu et al., 2006). However, the effects of cinaciguat on the DA are still unknown.

In this study, we first demonstrated *in vivo* that cinaciguat can preserve DA patency in neonatal rats. Since DA patency can be caused by vasodilatation and anti-remodeling, we further investigated these effects on isolated DA rings and by histochemical stains, respectively. To elucidate the effects of vasodilatation, we conducted an *ex vivo* tension study on DA tissue, and confirmed that cinaciguat dilated the oxygen-constricted DA, and this effect was blunted by the PKG inhibitor KT5823. In the histochemical stains, we found that cinaciguat could mitigate the intimal thickening and maintained DA luminal patency, which was associated with preserved cGMP expression. Taken together, these findings demonstrate that cinaciguat can prevent DA closure by the cGMP/PKG pathway. Previously, we have shown that BNP can prevent DA closure through the cGMP/PKG pathway, which is mediated by particulate GC (pGC) (Yeh et al., 2018). In parallel, this study further shows that activation of the sGC/cGMP pathway can also prevent DA closure by vasodilatation and anti-remodeling.

Proliferation and migration of SMCs are both important factors mediating vascular remodeling. In an animal model of vascular injury, cinaciguat augmented PKG signaling and prevented neointimal formation *via* decreasing SMC proliferation and migration (Hirschberg et al., 2013). To further determine the mechanisms of the anti-remodeling effect of cinaciguat, we investigated the effects of cinaciguat on DASMC proliferation and migration. We found that cinaciguat inhibited DASMC proliferation and migration induced by Ang II. These results were in line with the previous study showing that through the cGMP pathway, BNP inhibited DA and PA SMC proliferation and migration (Hsu et al., 2014; Yeh et al., 2018).

Reactive oxygen species production is essential in vascular remodeling of pulmonary hypertension and appears to be critical for SMC proliferation and migration (Fulton et al., 2017). Furthermore, mitochondria are one of the major sources of ROS (Turrens, 2003), so we examined mitochondrial ROS of DASMCs treated with cinaciguat to study the mechanism underlying the anti-proliferative and ant-migratory effects of cinaciguat. The results demonstrated that cinaciguat attenuated mitochondrial ROS production induced by Ang II. It is known that NADPH oxidase is a classical target of Ang II. However, emerging studies have shown that mitochondria, with crosstalk to NADPH oxidase, is also an essential source of Ang II-mediated ROS in VSMCs (Tsai et al., 2016; Fukai and Ushio-Fukai, 2020). In addition, mitochondria-derived ROS has been recently demonstrated with a key role during DA constriction (Michelakis et al., 2002; Turrens, 2003; Hong et al., 2014). Therefore, instead of NADPH oxidase, in this study, we investigated mitochondria ROS and revealed its role in DASMCs.

The final study was to examine the possible roles of signal transduction involved with anti-proliferative and anti-migratory effects of cinaciguat. It is known that the MAPK family and Akt signaling are both down-stream mediators of ROS, participating in SMC proliferation and migration (Muslin, 2008; Abeyrathna and Su, 2015). In addition, activation of the cGMP pathway inhibited MAPK and Akt signaling (Bouallegue et al., 2007; Wu et al., 2016). In this study, we found that cinaciguat inhibited Ang II-induced phosphorylation of the MAPK family and Akt dose-dependently, which was possibly because of its effect of enhancing cGMP signaling.

There are some limitations of this study. We did not examine if inhibitors of ERK, JNK, p38, and Akt could attenuate the effects of cinaciguat on DA regulation. In addition, we did not investigate the role of NADPH oxidase, which is a classical target of Ang II and a major source of ROS in VSMC. Thus, these important issues warrant further investigations.

In conclusion, cinaciguat prevents postnatal DA closure by vasodilation and anti-remodeling through the cGMP/PKG pathway. The mechanisms underlying anti-remodeling effects include anti-proliferation and anti-migration, with attenuation of mitochondrial ROS production, MAPKs, and Akt signaling. Thus, this study implicates that sGC activation may be a promising novel strategy to regulate DA patency.

## Data Availability Statement

The original contributions presented in the study are included in the article/Supplementary Material, further inquiries can be directed to the corresponding author/s.

## Ethics Statement

The animal study was reviewed and approved by Animal Care and Use Committee of the Kaohsiung Medical University. The Institutional Animal Care and Use Committee (IACUC) number is 102094. Animals were cared for in compliance with the guiding principles of the National Institutes of Health of the United States.

## Author Contributions

Y-CH wrote the manuscript. J-LY initiated the constructs and validated them, and supervised the experiments. Y-CL and B-NW designed the myograph experiments. J-HH designed the experiments and revised the manuscript. All authors have read and agreed to the published version of the manuscript.

## Conflict of Interest

The authors declare that the research was conducted in the absence of any commercial or financial relationships that could be construed as a potential conflict of interest.

## Publisher’s Note

All claims expressed in this article are solely those of the authors and do not necessarily represent those of their affiliated organizations, or those of the publisher, the editors and the reviewers. Any product that may be evaluated in this article, or claim that may be made by its manufacturer, is not guaranteed or endorsed by the publisher.
